# Feasibility and Safety of Intraoperative Radiotherapy with Low Energy X-ray Photon Therapy for Recurrent Gynecological Cancer: A Case Series

**DOI:** 10.3390/life12050685

**Published:** 2022-05-05

**Authors:** Hui-Hua Chen, Pei-Yu Hou, Wan-Hua Ting, Pei-Wei Shueng, Sheng-Mou Hsiao

**Affiliations:** 1 Department of Obstetrics and Gynecology, Far Eastern Memorial Hospital, Banqiao, New Taipei 220216, Taiwan; thandaaye24@gmail.com (H.-H.C.); stellatingwh@yahoo.com (W.-H.T.); 2 Department of Radiation Oncology, Far Eastern Memorial Hospital, Banqiao, New Taipei 220216, Taiwan; jcgv03@gmail.com; 3 Department of Industrial Management, Asia Eastern University of Science and Technology, New Taipei 220303, Taiwan; 4 Faculty of Medicine, School of Medicine, National Yang Ming Chiao Tung University, Taipei 112304, Taiwan; 5 Department of Radiation Oncology, Tri-Service General Hospital, National Defense Medical Center, Taipei 114202, Taiwan; 6 Graduate School of Biotechnology and Bioengineering, Yuan Ze University, Taoyuan 320315, Taiwan; 7 Department of Obstetrics and Gynecology, National Taiwan University College of Medicine and Hospital, Taipei 100226, Taiwan

**Keywords:** radiotherapy, genital neoplasms, female, surgical procedures, operative

## Abstract

Objectives: To evaluate the feasibility and safety of low energy X-ray photon intraoperative radiotherapy (IORT) as an adjuvant therapy for recurrent gynecological cancer.Methods: Medical records of all recurrence gynecological cancer patients who underwent IORT were reviewed. Results: Between January 2018 and December 2021, five women (including cervical cancer (*n* = 2), endometrial cancer (*n* = 2), and uterine leiomyosarcoma (*n* = 1)), who underwent IORT and surgical resection for recurrent gynecologic cancer were reviewed. A median dose of 15.62 Gy (range, 12 to 20 Gy) was used for IORT. Repeated IORT and surgical resection was performed in two women. Three women experienced local recurrence, and three women died during follow-up. The 1-year local control rate was 60%. The 2-year overall survival rate was 30%. There was no Clavien–Dindo classification grade III–V complication. Conclusion: IORT using low energy X-ray photon therapy seems to be feasible and safe as an adjuvant therapy in women who underwent salvage surgery for recurrent gynecologic cancer. However, large-scale prospective studies are needed to confirm our findings and evaluate its efficacy.

## 1. Introduction

The management of recurrent cervical and endometrial cancers can be challenging for the gynecologic oncologist. For resectable lesions, surgery is the first-line therapy for cancer recurrence. Post-operative radiation therapy has the role of prevention of local tumor recurrence or local control of the tumor, especially for R1 or R2 resection. However, those patients with tumor recurrence frequently have histories of previous irradiation with either external beam radiation or brachytherapy as their primary frontline treatment or adjuvant treatment in women with cervical cancer or endometrial cancer. Thus, the issue of re-irradiation for the recurrence disease to achieve disease control and avoid surrounding normal structure injury is a problem to be overcome. Intraoperative radiation therapy (IORT) is a special useful technique to deliver high doses to a focused region, especially for recurrent disease within the prior irradiation area. Owing to its nature of direct contact radiation to the tumor [1] and the ability of protection after proper packing, IORT allows maximal tumor control with irradiation and minimizing the radiation exposure of surrounding normal structures [1].

IORT can be delivered via different techniques, including intraoperative electron beam radiotherapy (IOERT) [2,3], high-dose-rate intraoperative brachytherapy (HDR-IORT) [4], and low energy X-ray photon IORT with cone (spherical), balloon, or catheter applicators of variable sizes implanted during the surgery to match the defined tumor bed or region at risk. Each technique has its unique method and suitable indication.

For IOERT, from when it was introduced in the 1960s, patients need to be transported from the operation room to the radiation department [5]. In addition, the mobile linear accelerator machine and the less self-shielding demanded of IOERT provide a more flexible use for intraoperative electron radiotherapy [1,2,3,6,7,8]; however, the cone applicator of IOERT is rigid and might be difficult to fit to the irregular shape or narrow cavity of the tumor bed or region at risk in the pelvic site. Thus, the use of IOERT for the treatment of recurrent gynecological malignancies is limited.

The issue of shielding demanded in the operation room is not solved for HDR-IORT, and the relatively prolonged treatment time is also another problem for HDR-IORT.

**Figure 1:**
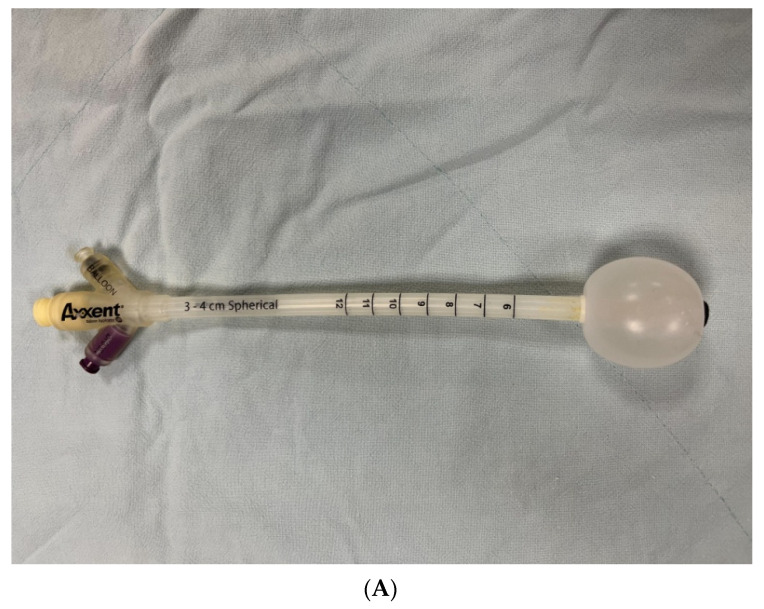

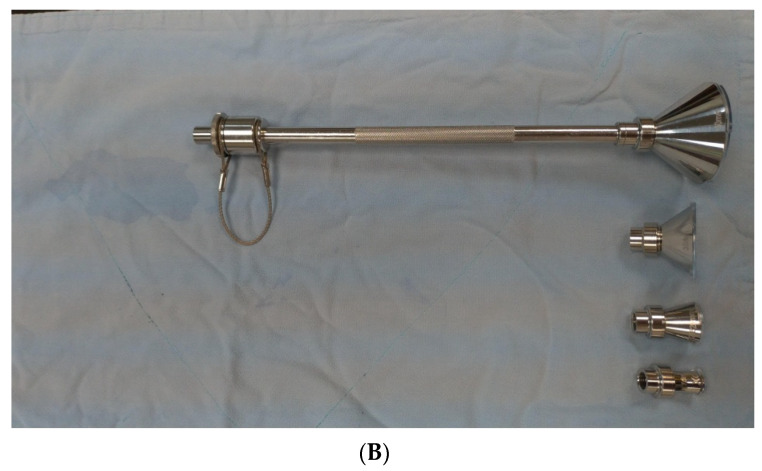
(**A**) Balloon and (**B**) cone applicators.

The Axxent Electronic Brachytherapy System (Xoft Inc., Fremont, CA, USA) [9,10,11,12] and Intrabeam (Carl Zeiss AG, Jena, Germany) [13] are both commonly used with low energy X-ray photon IORT. The mobile machines and less shielding requirement in the operation room have made them popular in IORT applications. They have different sized balloon (Figure 1A) and cone (Figure 1B) applicators that can be suited for a wide range of target volumes and shapes. They have the advantage of dose distribution with steep dose gradients. The limited dose penetration within 0.5–1 cm can provide safe treatment without excessive surrounding normal tissue damage and can prevent related toxicity.

The Axxent Electronic Brachytherapy System has been used for breast cancer patients [9,10,11,12]. It delivers a single high dose to the focused area and has a limited depth with a low energy 50 kv X-ray to perform effective IORT treatment, reduces exposure to surrounding normal tissue, and minimizes side effects. The Axxent Electronic Brachytherapy System has been used as adjuvant brachytherapy for early stage endometrial cancer in women with a high risk for recurrence [14]. A total of 15 patients with stage I or II endometrial cancer were enrolled. There were only four grade I and 2 grade II brachytherapy-related adverse events. The grade I adverse events included dysuria, vaginal dryness, mucosal atrophy, and rectal bleeding. The grade II adverse events included dysuria and vaginal pain. There were no grade III or IV adverse events. The Axxent Electronic Brachytherapy System seemed to perform well and was associated with limited toxicity [14].

To our knowledge, clinical use of the Axxent Electronic Brachytherapy System in the treatment of recurrent gynecologic cancers has never been reported. Thus, this retrospective study aims to evaluate the feasibility and safety of low energy X-ray photon IORT with the Axxent Electronic Brachytherapy System as an adjuvant therapy in women who have undergone salvage surgery for recurrent gynecologic cancer.

## 2. Materials and Methods

Medical records of women with recurrence of cervical cancer, endometrial cancer, or uterine leiomyosarcoma, and undergoing salvage surgery and low energy X-ray photon IORT in a tertiary referral hospital, were reviewed. The study was approved by the Research Ethics Review Committee of the hospital (No.110259-E).

IORT with 50 kv X-ray photons (Axxent Electronic Brachytherapy System, Xoft Inc., Fremont, CA, USA) was delivered by variable sized cone or balloon applicators limited to the at-risk region and depth to maximize dose to the target and minimize exposure to the surrounding normal tissues. The commonly used radiation dose of IORT was ranged within 10–20 Gy in a single fraction. The actual delivering dose depended on the treatment site, surrounding tissue/organ tolerance, margin status of gross residual or microscopic residual disease, and the treatment goals of adjuvant, salvage, or palliative settings. The use of cone or balloon applicators depended on the shape of the treatment regions. The cone applicators were for the relatively flat region, and the balloon applicators were for the spherical region. Many different sizes of applicators can be used for different treatment volumes. The aim was to match the tumor bed or region at risk volume to the applicators without attenuation radiation dose in the gap. Thus, the curative high dose IORT was delivered to the surface with limited penetration depth to obtain the therapeutic effect. Concurrently, the use of several applicators were allowed dependent on the physician’s choice. Cancer recurrence was assessed according to the appearance of abnormal radiological findings or histological proof from biopsy analyses, whichever occurred first. Recurrence-free survival was measured from the date of primary surgery, completion of definitive radiotherapy or salvage surgery to the date of clinically defined recurrence, disease progression, or the last follow-up visit. Overall survival was calculated from the date of salvage surgery to the date of death from any cause or the last follow-up visit. Stata version 11.0 (Stata Corp, College Station, TX, USA) was used for statistical analyses. Survival curves were generated using the Kaplan–Meier method.

## 3. Results

Between January 2018 and December 2021, there were five women who underwent eight procedures of IORT for recurrent gynecologic cancers including cervical cancer, endometrial cancer, and uterine leiomyosarcoma. Baseline data were shown in Table 1.The IORT procedure was performed after salvage debulking surgery for cancer recurrence. Doses of IORT ranged from 12 to 20 Gy (median, 15.62 Gy) using 50 kv photons (Table 2). Dosage was prescribed at the surface of the cone or balloon applicator on the pelvic side wall, pelvic lymph node region, abdominal wall, or vaginal stump, respectively.

**Figure 2:**
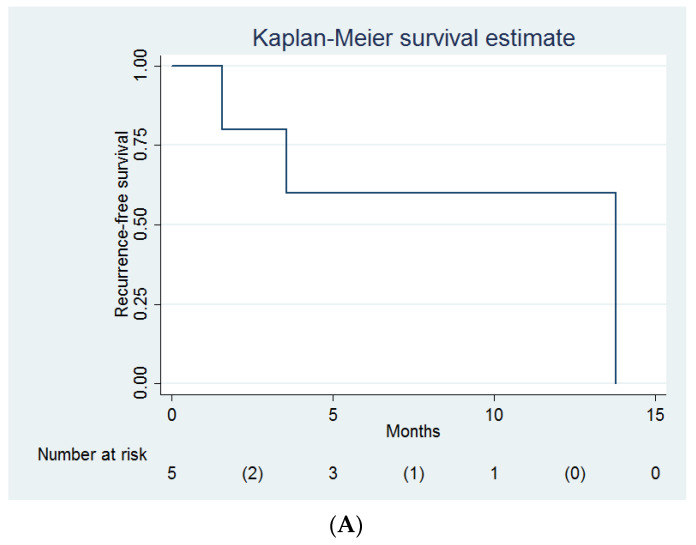

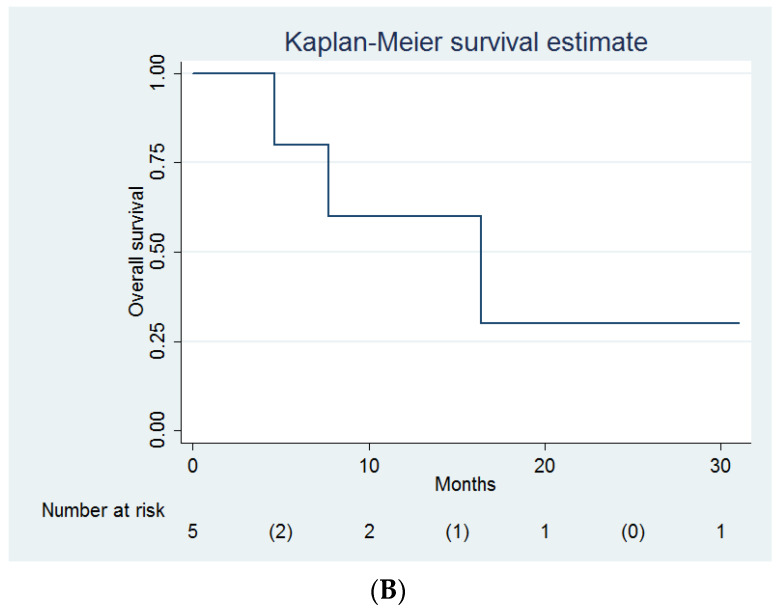
(**A**) Recurrence-free survival and (**B**) overall survival of women undergoing intraoperative radiotherapy (*n* = 5).

Three women experienced local recurrence (Figure 2A), and three women died during follow-up (Figure 2B). The overall local control rate was 40% (2/5), and the overall survival rate was 40% (2/5). The median recurrence-free survival was 13.8 months (95% confidence interval (CI) = 1.6 months to infinity), and the median overall survival was 16.4 months (95% CI = 4.7 months to infinity). The 1-year local control rate was 60% (95% CI = 13% to 88%). The 2-year overall survival rate was 30% (95% CI = 1% to 72%). However, there was no Clavien–Dindo classification grade III–V complication.

Both endometrial cancer patients were treated with optimal debulking surgery and IORT, and both women did not have any local recurrence after treatment. However, one woman with endometrial cancer died owing to lung and liver metastasis (Table 2).

Repeated IORT procedures were performed in two women (Patients no. 2 and 5, Table 3). The woman who underwent pelvic exenteration and IORT for recurrent cervical cancer, experienced vaginal stump recurrence 17 months after exenteration. The woman with recurrent uterine leiomyosarcoma experienced repeated recurrence and died from recurrence despite repeated optimal debulking surgeries and IORT; however, the recurrent sites did not locate at prior IORT sites.

## 4. Discussion

In our study, all women underwent IORT procedures at the end of salvage surgery (Table 2 and Table 3). In our case series, with the aid of IORT cone and balloon applicators (Figure 1a,b), low energy X-ray photon IORT with the Axxent Electronic Brachytherapy system seemed to be feasible as an adjuvant therapy in women who had undergone salvage debulking surgery for recurrent gynecologic cancers, even in women with vaginal stump, pelvic side wall, and abdominal wall metastasis (Table 2 and Table 3).

Common treatment-related toxicities of IOERT and HDR-IORT include peripheral neuropathy, gastrointestinal fistula, gastrointestinal perforation, gastrointestinal obstruction, ureteral stricture, persistent pain, pelvic abscess, perioperative hemorrhage, delayed wound healing, and lymphedema [1,2,15,16,17,18,19,20,21]. In our study, there were no Clavien–Dindo classification grade III–V complications (Table 2), even in women who received repeated IORT (Table 3). Thus, the use of low energy X-ray photon IORT using 50 kv photons to the highly focused area seems to be safe for women who have undergone salvage surgery for recurrent gynecologic cancer patients.

In this study, women who underwent low energy X-ray photon IORT included those with recurrent cervical cancer, recurrent endometrial cancer, and recurrent uterine leiomyosarcoma (Table 2 and Table 3). IORT has been used as a boosting technique to deliver the irradiation directly to the resection bed. The technique is usually used in women with cervical cancer recurrence. The addition of IORT increases the local control rate of recurrent cervical cancer [22]. The role of IORT in endometrial cancer is less frequently reported. Both of our endometrial cancer patients underwent an optimal salvage debulking operation and had a good local control. Dowdy et al. also reported a local control rate of 84% in women with no residual tumor and 47% in women with residual tumors [18]. The amount of residual tumor is significantly associated with a local control rate after IORT [18,23]. Arians et al. reported that the 5-year overall survival rate was 50% in recurrent endometrial carcinoma [3]. Thus, IORT seems to be promising for the treatment of recurrent endometrial cancer patients.

In our patient, repeated recurrent uterine leiomyosarcoma was noted despite the use of IORT. However, the recurrent sites after IORT were not located at previous IORT sites (Table 1 and Table 2). The aggressive behavior of recurrent uterine leiomyosarcoma may increase the failure rate. Coelho et al. also reported that the overall survival was inferior in the sarcoma group compared with the epithelial group (i.e., the 2-year, 5-year, and 8-year overall survival rates were 60%, 20%, and 6% in the sarcoma group versus the 2-year, 5-year, and 8-year overall survival rates of 73%, 57%, and 40% in the epithelial tumor group, respectively, *p* = 0.005) [24]. However, many patients achieve long-term overall survival and local control without significant morbidity after salvage surgery and IORT, especially in the case of clear margins [24]. Barney et al. also reported a good local control rate in women with primary or locoregionally recurrent uterine sarcoma who received adjuvant IORT after surgical resection [25].

The initial clinical experience of IORT was for the treatment of cervical cancer. Nonetheless, most case series of IORT in gynecological cancer management were for recurrent cervical, endometrial, vaginal, vulvar, and ovarian cancer [2,3,8,15]. Considering the characteristic of IORT for delivering a large irradiation dose and limiting the irradiation exposure to the adjacent regions, IORT is especially suitable for tumors in abdominopelvic regions with much irradiation susceptibility and normal organs surrounding it.

Following the evolution of radiation techniques, the application of IORT has been developed and used for several tumors in different regions and various settings in recurrent or primary cancer management. This includes early breast cancer [26,27,28], recurrent head and neck cancer [29,30,31], locally advanced or recurrent colorectal cancer [32,33,34,35], extremity soft tissue sarcoma [36,37], retroperitoneal sarcoma [38,39], pediatric tumors [40], genitourinary cancers (such as bladder cancer [41], renal cell carcinoma [42], or prostate cancer [43]), gastric cancer [44,45], or pancreatic cancer [46,47,48]. Most of the reported data indicate the above cancer treatments were delivered with IOERT. Some of them were treated with HDR-IORT. The variety of clinical indications of IORT has provided a growing number of patients who have received this radiation modality.

According to the previously published clinical series of recurrent gynecological malignancies, IORT was delivered with electrons in the majority, and others were delivered with HDR-IORT. Surgical margin status plays an important role in the clinical outcome of recurrent gynecologic cancers treated with IORT. Dowdy et al. reported that salvage surgery with negative surgical margins combined with IORT could provide good long-term local control and survival (the 5-year overall survival rate was 71% in R0 surgical resection, compared with the 5-year overall survival rate of 47% in R1 surgical resection and the 5-year overall survival rate of 0% in R2 surgical resection) [18]. Similarly, Delara et al. reported that the 3-year progression-free survival rates were 51.8% in negative margins, 20.5% in the microscopic margin, and 0% in the macroscopic surgical margin [17]. Martínez-Monge et al. reported a poor local control rate in women with recurrent cervical cancer with positive lymph nodes, parametrial involvement, and/or incomplete resection despite the use of IORT [49].

The Memorial Sloan-Kettering Cancer Center has reported their experience of a combination of surgical resection and HDR-IORT for treating recurrent gynecologic cancers, including cervical (*n* = 9), uterine (*n* = 7), and vaginal cancer (*n* = 1) [50]. The 3-year local control rate was 67%, and the 3-year overall survival rate was 54%. However, the 3-year local control rate was only 25% in women with gross residual tumor, compared with 83% in women with complete gross resection (*p*< 0.01); thus, they emphasized that patient selection is very important for treating recurrent gynecological cancers with a combination of surgical resection and IORT [50]. In contrast to our report, gastrointestinal obstruction (*n* = 4), abscess (*n* = 3), peripheral neuropathy (*n* = 3), rectovaginal fistula (*n* = 2), and uretheral obstruction (*n* = 2) were observed in some patients [50].

According to the successful experience of low energy X-ray photon IORT for early breast cancer [9,10,11,12], an undetermined issue is the feasibility of low energy X-ray photon IORT for treating recurrent gynecologic cancers. Shielding in the operation room is demanded while performing IOERT or HDR-IORT. However, shielding is needed less for delivering low energy X-ray photon IORT (i.e., Axxent Electronic Brachytherapy System or Intrabeam System).In addition, the machine for delivering low energy X-ray photon IORT is easily moved and can be transported to different operation rooms or even to different hospitals. Its cost-effectiveness and feasibility have made the low energy X-ray photon IORT system increasingly popular in clinical practice.

The Axxent Electronic Brachytherapy System has been used for treating breast cancer patients [9,10,11,12]. In our institution, IORT was delivered with the Axxent Electronic Brachytherapy System. The local control when using the Axxent Electronic Brachytherapy System to treat early breast cancer was reported to be similar to the conventional external beam radiation therapy [9,10,11,12]. In our study, the use of the Axxent Electronic Brachytherapy System in the treatment of recurrent gynecological cancer is an innovative approach. Our data provide detailed experience and clinical outcomes of delivering IORT with the Axxent Electronic Brachytherapy System in patients with recurrent gynecological cancers, including cervical cancer, endometrial cancer, and uterine leiomyosarcoma. Thus, our results can be considered as an important reference for pretreatment consultation for those women scheduled for salvage surgical resection of the above gynecological cancers.

In addition to recurrent cervical cancer, IORT might have a role as the adjuvant therapy after primary surgical resection of stage IIB cervical cancer. A 5-year survival rate of 49–88%, a 5-year disease-free survival of 70%, and a local control rate of 79–100% have been reported in women with stage IIB cervical cancer receiving surgical resection and IORT [51,52,53]. Similarly, the 2-year disease-free survival in stage IIB cervical cancer treated by concurrent chemoradiotherapy is estimated as 75% [54]. Gao et al. reported the clinical outcome of adjuvant IORT in women with stage IIB cervical adenocarcinoma [52]. After a total dose of weekly intracavitary brachytherapy (total dose HDR 12–14 point A), an IORT dose of 18–20 Gy was delivered using a 12 MeV electron beam during hysterectomy. Adjuvant chemotherapy with 4–6 cycles of cisplatin and 5FU was also performed. The 5-year overall survival and disease-free survival rates were 77.8% and 70.4%, respectively. Local control in the field was 100% even with close/positive resection margins [52]. Gao et al. concluded that IORT was safe and feasible and appeared to confer a disease control benefit to surgical resection, especially for cervical adenocarcinoma [52]. Future researches may assess the effect of the Axxent Electronic Brachytherapy System as an adjuvant IORT after primary surgical resection in women with stage IIB cervical adenocarcinoma.

Gynecologic cancer is a very stressful experience for women and treatment can compromise fertility and reproductive capacity [55]. In addition, therapy for recurrent gynecologic cancers may have a detrimental effect on patients’ quality of life, psychological distress, and even sexual function [55,56]. For example, vaginal vault resection is an effective treatment for vaginal recurrence of cervical cancer; however, the complications of this resection procedure led to a reduced quality of life [57]. To our knowledge, there is no study mentioning about the impact of IORT on quality of life. Future researches can explore its detailed impact on quality of life.

Our study is limited due to its retrospective nature, small sample size, and heterogeneous cancer type. Future large-scale studies are needed to prove the efficacy and safety of low energy X-ray photon IORT for women undergoing salvage surgical resection of recurrent gynecologic cancers.

## 5. Conclusions

IORT using low energy X-ray photon therapy seems to be feasible and safe as an adjuvant therapy in women who have undergone salvage surgery for recurrent gynecologic cancer. However, large-scale prospective studies are needed to confirm our findings and evaluate its efficacy.

## Figures and Tables

**Table 1:** Baseline data of cancer patients (*n* = 5).

Case No.	Age (Years)	Type of Cancer	FIGO Stage	Primary Treatment	RFS † (Months)
1.	62	Endometrial cancer, endometriod cell, grade 1	IA	Staging surgery	40.4
2.	71	Cervical cancer, gastric type mucionous carcinoma	IB1	Hysterectomy + CCRT	28.7
3.	65	Endometrial cancer, carcinosarcoma	IB	Staging surgery + sandwich chemoradiotherapy	11.5
4.	53	Cervical cancer, squamous cell carcinoma, grade 2	IIIB	CCRT	3
5.	42	Uterine leiomyosarcoma	IIB	Debulking operation	2.8

CCRT = concurrent chemoradiotherapy; FIGO = International Federation of Gynecology and Obstetrics; RFS = recurrence-free survival; † RFS was measured from the date of primary surgery or completion of CCRT to the date of recurrence.

**Table 2:** Clinical outcome of women who underwent intraoperative radiotherapy (*n* = 5).

Case No.	Type of Cancer	Optimal Debulking Surgery	IORT Site and Dose	IORT Applicator (Cone Diameter cm/Balloon mL)	Grade III–V Complication †	Local Recurrence or Progression	Death	RFS ‡ (Months)	OS § (Months)
1.	Endometrial cancer	Yes	16 Gy to left pelvic wall	Balloon 25 mL	No	No	No	9.5	9.5
2.	Cervical cancer	Yes	12 Gy to vaginal stump	Cone diameter 3.5 cm	No	Yes	No	13.8	31.0
3.	Endometrial cancer	Yes	16 Gy to left pelvic wall and 12 Gy to vaginal stump	Balloon 50 mL for left pelvic wall and balloon 30 mL for vaginal stump	No	No	Yes	7.8	7.8
4.	Cervical cancer	No	15 Gy to left pelvic lymph node	Balloon 20 mL	No	Yes	Yes	3.6	4.7
5.	Uterine leiomyosarcoma	Yes	20 Gy to vaginal stump	Balloon 30 mL	No	Yes	Yes	1.6	16.4

IORT = intraoperative radiotherapy; OS = overall survival; RFS = recurrence-free survival. † Complication was evaluated with the Clavien–Dindo classification. ‡ RFS was measured from the date of IORT to the date of recurrence, the last follow-up visit, or death. § OS was measured from the date of IORT to the date of the last follow-up visit, or death.

**Table 3:** Clinical outcome of women who underwent repeated intraoperative radiotherapy (*n* = 2).

Case No.	Type of Cancer	Optimal Debulking Surgery	IORT Site and Dose	IORT Applicator (Cone Diameter cm/Balloon mL)	Grade III–V Complication	Local Recurrence	Death	RFS ‡ (Months)	OS § (Months)
2.	Cervical cancer	Yes (pelvic exenteration)	16 Gy to vaginal stump	Balloon 25 mL	No	Yes	No	17.1	17.1
5.	Uterine leiomyosarcoma	Yes	17.88 Gy to left upper quadrant abdominal wall and 16 Gy to suprapubis	Both with cone diameter 5 cm	No	Yes	Yes	3.1	11.3
5.	Uterine leiomyosarcoma	Yes	16 Gy to left pararectum and 15 Gy to left and right lower abdominal wall	Balloon 30 mL for left pararectum, simultaneously use two cone applicators with diameter 5 cm without overlap or gap for left abdominal wall and cone diameter 5 cm for right abdominal wall	No	Yes	Yes	1.1	8.0

IORT = intraoperative radiotherapy; OS = overall survival; RFS = recurrence-free survival. ‡ RFS was measured from the date of repeated IORT to the date of local recurrence, the last follow-up visit, or death. § OS was measured from the date of repeated IORT to the date of the last follow-up visit, or death.

## Data Availability

The data presented in this study are available on reasonable request to the corresponding author.
